# High-Performance PET-TM/PTFE-like Composite Membranes for Efficient Salt Rejection via Air Gap Membrane Distillation

**DOI:** 10.3390/polym17030290

**Published:** 2025-01-23

**Authors:** Veronica Satulu, Liubov I. Kravets, Oleg L. Orelovich, Bogdana Mitu, Gheorghe Dinescu

**Affiliations:** 1 National Institute for Laser, Plasma and Radiation Physics, 077125 Magurele, Romania; veronica.satulu@inflpr.ro; 2 Flerov Laboratory of Nuclear Reactions, Joint Institute for Nuclear Research, 141980 Dubna, Russia; kravets@jinr.ru (L.I.K.); orel@jinr.ru (O.L.O.)

**Keywords:** PET-TM, PTFE, TFC membranes, RF magnetron sputtering, salt rejection, AGMD

## Abstract

The global water scarcity crisis requires urgent action to improve wastewater treatment and develop sustainable water resources. This study focuses on producing Thin Film Composite (TFC) membranes based on polyethylene terephthalate track membranes (PET-TM) coated with polytetrafluorethylene-like material (PTFE), named PET-TM/PTFE-like, designed to purify saline water using Air Gap Membrane Distillation (AGMD) technique. The research emphasizes the optimization of these membranes’ chemical composition and surface characteristics by plasma that enhances their hydrophobicity and overall operational efficiency. A systematic investigation was conducted to clarify the relationship between water flux and salt rejection, enabling the customization of membrane properties for better performance. It was shown that salt rejection exceeding 99% is obtained for all the investigated PET-TM/PTFE-like membranes, with values up to 99.63% for the PET-TM(250 nm)/PTFE-like(200 nm) system and condensate flows as high as 1325 g/m^2^h for the PET-TM(450 nm)/PTFE-like(200 nm) system. This comprehensive analysis identified the most effective TFC configurations for AGMD applications, providing a promising pathway to advance desalination techniques and wastewater treatment solutions.

## 1. Introduction

Water scarcity is one of the most critical global challenges, with severe shortages projected to affect billions of people by 2025 [1,2,3]. Freshwater consumption is dominated by agriculture, which utilizes approximately 70% of available resources, followed by industrial (19%) and domestic (11%) uses [4]. This crisis is exacerbated by factors such as overexploitation, pollution, poor management practices, climate change, and rapid population growth. According to the United Nations, approximately 2 billion people currently live in regions experiencing physical water scarcity, with an additional 500 million approaching this threshold. Furthermore, an estimated 1.6 billion individuals face economic water scarcity, where financial constraints and inadequate infrastructure hinder access to clean and sufficient water supplies [5]. Innovative water management technologies are essential to addressing this escalating crisis. Among the available solutions, membrane separation processes have emerged as effective and versatile tools for water and wastewater treatment. These systems provide significant advantages over traditional methods, including lower energy consumption, compact system designs, ease of operation, and the potential for material recovery [6]. Membrane Distillation (MD), a thermally driven separation process, has gained prominence for its ability to handle challenging feed waters such as seawater and industrial effluents [7]. MD achieves high rejection rates for non-volatile contaminants while operating at lower pressures and temperatures compared to conventional desalination technologies like traditional distillation process and/or reverse osmosis (RO) [8,9,10,11,12]. Within MD configurations, Air Gap Membrane Distillation (AGMD) is particularly noteworthy [13] since the incorporation of an insulating air gap between the membrane and the condensation surface enhances evaporation efficiency and salt rejection while reducing thermal conductivity and heat loss [14,15]. Despite these advantages, membrane fouling remains a significant barrier to the broader adoption of MD and other membrane technologies. Crystalline, organic, particulate, colloidal, and microbial fouling can reduce the water flux and compromise the long-term performance of such systems [16]. This underscores the need for membranes with high water flux [17], effective rejection capabilities, robust stability, and low fouling potential [18,19,20]. Traditional fabrication methods, such as phase inversion [21,22,23], have limitations, including low structural precision and lengthy processing times [24]. To address these challenges, Track-Etched Membranes (TMs) offer a promising alternative [25,26] as the preparation method allows precise control over pore size (from nano- to microscale) and pore morphology (e.g., cylindrical, conical, funnel-like, or cigar-like). This high degree of customizability enhances transport and retention characteristics, making TMs suitable for diverse applications, from industrial processes to municipal water treatment [27]. Further advancements in membrane technology have led to the development of thin-film composite (TFC) membranes, which integrate an ultrathin active layer with a porous support matrix. This design enhances separation performance by utilizing the active layer for solute solubilization and diffusion while the porous support maintains structural integrity [28]. Fluoropolymer thin films, such as polytetrafluoroethylene (PTFE), have been identified as ideal candidates for these membranes due to their exceptional chemical, thermal, and oxidative stability [29,30]. Composite membranes like PET-TM/PTFE ones (Polyethylene Terephthalate, track-etched membranes with polytetrafluorethylene) exemplify this coaction by combining PTFE’s hydrophobic and anti-fouling properties with the mechanical strength and durability of PET-TMs. Although PET-TMs are inherently hydrophilic, the application of PTFE coatings imparts critical water resistance, enabling better control over wetting properties, permeability, and anti-clogging performance. Various thin-film deposition techniques have been explored to produce these advanced membranes, each with unique advantages and limitations [31]. Several advanced techniques are used to deposit thin films from solid fluorine-containing materials, and in particular for PTFE. These include electron beam irradiation [32,33], thermal chemical vapor deposition [34,35], and vacuum evaporation [36,37]. Generally, these methods are suitable for low molecular weight polymers that can evaporate and condense onto substrates without undergoing chemical reactions. However, the high temperatures required for evaporation often cause most polymers to fragment, forming gaseous fragments. These fragments condense on the substrate as isolated particles rather than forming a cohesive polymer network [38]. An alternative approach is laser ablation, which utilizes solid targets [39,40]. However, this method is limited by small deposition areas and challenges in achieving film uniformity. A more innovative technique is radiofrequency magnetron sputtering [41,42,43,44,45,46]. In this approach, volatile fragments sputtered from a polymeric target enter the plasma phase, acting as gaseous precursors for polymerization directly on the substrate surface. This technique offers several advantages, including improved uniformity of the thin films and greater control over their properties [38,47].

By connecting these advanced deposition techniques and novel materials, ongoing research aims to push the boundaries of membrane technologies. This approach is critical for addressing global water scarcity and wastewater treatment challenges, ultimately securing sustainable water resources for the future. While our previous studies were focused on the obtaining of various types of thin film composite membranes for obtaining asymmetry of conductivity [48], antimicrobial and antiadhesive fluorocarbon-based TFC [49] or combined hydrophilic/hydrophobic membranes based on PET-TM/PTFE-like thin films [50], this study focuses on developing PET-TM/PTFE-like Thin Film Composite (TFC) membranes specifically designed for saline water purification via Air Gap Membrane Distillation, by using deposition of thin layers of PTFE-like material with thicknesses below that of the initial effective pores diameters on PET-TM membranes. The primary objective is to optimize the chemical composition and surface properties of the TFC membranes to enhance their hydrophobicity, thereby improving overall performance. The effects of plasma deposition and treatment conditions on the membranes’ topographical, structural, and chemical properties are systematically analyzed, enabling the identification of optimal configurations. The study investigates the interplay between water flux and salt rejection upon tailoring the membrane characteristics via the thin PTFE-like layer in order to establish the most effective PET-TM/PTFE-like TFC systems for AGMD applications.

## 2. Materials and Methods

### 2.1. PET-TM/PTFE-like TFC Membranes Synthesis

Track-etched polyethene terephthalate membranes (PET-TM) of 10 µm thickness, with cylindrical pores having effective pore diameters of 250 nm and 450 nm and a pore density of 2 × 10^8^ cm^−2^ and 6 × 10^7^ cm^−2^, respectively, were used as porous supports in this study. These membranes were selected for experiments in order to evaluate the effect of the initial pores’ diameter on the characteristics of the final TFC membranes produced in this work. To obtain the PET-TM membranes, the initial 10 μm thick commercial PET foil was irradiated with positively charged krypton ions accelerated on a cyclotron U-400 (JINR, Dubna, Russia) with an energy of ~3 MeV/nucleon. To form pores in the irradiated film, the exposed foils were subjected to chemical etching at 80 °C in an aqueous 6 mol/L sodium hydroxide (NaOH) solution for 2 min and, respectively, 4 min, according to the final desired dimension of pores of 250 and correspondingly 450 nm. Before etching, to increase the selectivity of the etching process along the ion tracks, the irradiated film was exposed to UV radiation from a source LE-30 lamp (LLC PCF “Polus”, Moscow, Russia) with a maximum intensity of the emission spectrum in the wavelength range of 280–315 nm. The technique to produce the track-etched membranes is described in more detail in [51]. The membrane samples were cut in circular form with a diameter of 90 mm before the experiments.

The magnetron targets were prepared from a PTFE rod (Goodfellow, Cambridge, UK) machined according to the technical specifications of the 1-inch magnetron sputtering source (diameter: 25.4 mm, thickness: 3.18 mm). They were cleaned by ultra-sonication in ethanol before mounting on the magnetron head and submitted to a pre-sputtering process prior to being used for deposition to clean the surface.

PTFE-like thin film deposition on the PET-TM porous support was conducted in a spherical stainless-steel vacuum chamber evacuated by a turbomolecular/rotary pumping system down to a base pressure of 1 × 10^−2^ Pa. A Pfeiffer gauge monitored the chamber’s pressure, and an electronic mass flow controller (Bronkhorst High-Tech B.V., Ruurlo, NL, USA) controlled the gas flow rates. The magnetron sputtering source (Kurt J. Lesker Company Ltd. Hastings, UK) is mounted on one port of the deposition vacuum chamber, positioned at 45° from the normal to the substrate and with the distance between the central point of the target to the central point of the substrate holder of 6 cm. A detailed description of the deposition setup is provided in [52]. Under an RF power of 80 W, 13.56 MHz (established by a Cesar power supply of Advanced Energy Industries U.K. Limited, London, UK) injected via an automatic matching box, and an Ar flow of 100 sccm setting a working pressure of ~0.15 Pa, the established deposition rate onto a flat substrate continuously rotating during the process with 200 rpm is around 4 nm/min. The results were determined by contact profilometry method upon using a Tenkor P6 profilometer on a PTFE-like coating deposited on a flat Si(100) substrate provisioned with an uncoated step. Deposition of thin films onto PET-TM membranes was conducted at room temperature at times that allowed the achievement of 100 nm and 200 nm for the membranes with initial pore diameters of 250 nm and, respectively, of 200 nm and 300 nm thickness for the membranes with initial pore diameters of 450 nm, corresponding in all cases to a partial covering of the membranes’ pores. We will show in the following that the obtained deposits have similarities with the original PTFE target, like hydrophobicity, while possessing at the same time a structure and chemical bonds apart from the original target, as in most of the polymeric thin films synthesized by plasma-driven processes. Therefore, throughout the entire manuscript, we refer to these deposits as PTFE-like material.

The side of the membrane with the PTFE-like deposit on it resulting from direct exposure to plasma is defined in the following as the *active side*, while the opposite side, which was in contact with the substrate holder during deposition, will be named the *back side.*

### 2.2. Membrane Characterization Techniques

Initial and PTFE-like-coated PET-TM membranes were characterized as described below.

Contact angle measurements were conducted at ambient temperature using a goniometer CAM 101 (KSV Instrument Ltd., Helsinki, Finland)incorporating a CCD camera. Manual deposition of 2 μL distilled water droplets onto the membrane surface enabled the measurement of contact angles, with each reported value representing an average derived from 10 individual droplets.

Scanning Electron Microscopy (SEM) was used to investigate the sample microstructure and evaluate the modification of pores geometry on the *active side* upon PTFE plasma deposition. SEM measurements were performed on a Hitachi SU-8020 (Hitachi High-Technologies, Ibaraki, Japan) ultra-high-resolution microscope operating at a low accelerating voltage (3 kV) to avoid damaging the polymeric samples. Before analyses, the composite membrane samples were covered by vacuum thermal evaporation with a thin gold layer deposited to prevent charge accumulation.

The chemical structure of the as-synthesized thin film membranes was analyzed using Fourier Transform Infrared Spectroscopy (FTIR) with a Bruker Vertex 70 spectrometer (Bruker, Billerica, MA, USA). The spectra were recorded over the range of 500–4000 cm^−1^, utilizing a resolution of 1 cm^−1^ and an average of 32 scans, with an attenuated total reflectance (ATR) module featuring a germanium crystal [53].

X-ray Photoelectron Spectroscopy (XPS) analyses were conducted using a K-Alpha Thermo Scientific ((ESCALAB™ XI+, East Grinstead, UK) spectrometer with a hemispherical analyzer. Photoelectron excitation was achieved through X-ray radiation from an aluminum anode (AlKα, 1486.6 eV) at a tube voltage of 12 kV and a current of 3 mA. Survey spectra were obtained to analyze the elemental composition of the porous composite membrane surface. In addition, high-resolution spectra for C1s, F1s, and O1s were recorded to examine the chemical bonding characteristics of the material.

The effective pore diameter was calculated from the experimental data with an accuracy of 3% using the Hagen–Poiseuille equation [54]. The calculations were based on air permeability data measured at a certain pressure gradient using a float-type flowmeter.

The values of liquid entry pressure (LEP_W_) for hydrophobic membranes, which is the minimum pressure of a liquid that is necessary for its penetration into membrane pores, were calculated by the Young–Laplace equation [55]

(1) $${\mathrm{LEP}}_{w}=\frac{-2B\cdot \gamma_{L}\cdot \;\cos\theta_{w}}{r_{\max}}$$

where *B* is a geometric factor that is governed by the membrane pores’ structure (for cylindrical pores, *B* = 1), γ_L_ is the water surface tension (N/m), *r*_max_ is the maximum pore size of the membrane, and θ_w_ is the water contact angle at the membrane surface.

### 2.3. Water Transport in Membrane Distillation Process

Studies on the Air Gap Membrane Distillation (AGMD) process were conducted to assess the efficacy of modified membranes for saline water desalination using a laboratory-scale vertical membrane distillation module (MDM). The membrane utilized had an active surface area of 5 × 10^−3^ m^2^. The AGMD process was performed under varying feed and coolant temperature conditions to evaluate performance metrics.

The MDM configuration was fabricated from polycaproamide (Caprolon, Smart Snab Co., Moscow, Russia) and designed to incorporate a fixed air gap of 4 mm. It is comprised of three distinct compartments: one designated for the feed solution, another for the coolant water, and a central permeation section responsible for collecting the permeate. The membrane, supported by a 2 mm thick perforated plate, was strategically placed between the feed and permeate compartments. As vapors from the feed solution permeated through the membrane pores, they entered the air gap and subsequently condensed on an aluminum coolant plate, which is in contact with the coolant water compartment. The feed solution, which had a concentration of 15 g/L NaCl, circulated in a closed-loop system between the MDM and a LOIP LT-100 liquid thermostat (LOIP LTD, Saint Petersburg, Russia) operational at 65 °C. On the other hand, the coolant water, maintained at a temperature of 10 °C, circulated between the MDM and an appropriate cooling apparatus. The choice of the temperature of the solution used and the water used to cool the solid partition, as well as the salt concentration in the solution, was based on an analysis of literature data, which are most completely presented in reviews [6,7].

**Figure 1:**
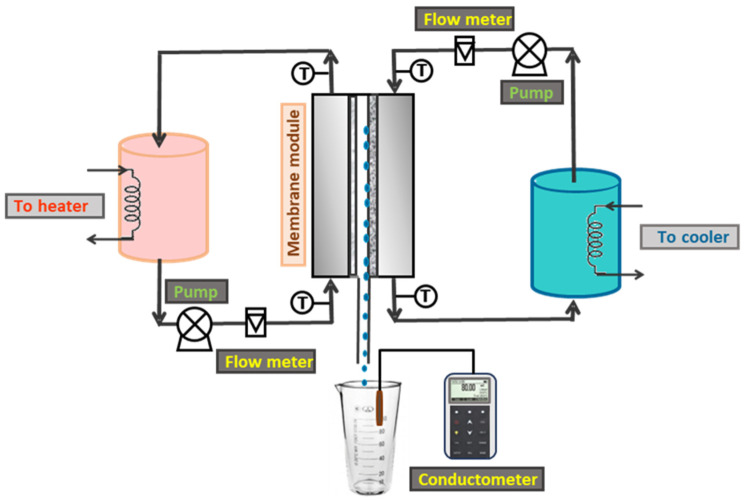
Schematical AGMD experimental setup.

The LOIP LT-100 liquid thermostat and LOIP FT-211-25 liquid cryostat (LOIP LTD, Saint Petersburg, Russia) were utilized for the investigation, with the temperatures of the feed and coolant monitored via electronic thermometers providing an accuracy of ±0.1 °C, as illustrated by ’T’ in the accompanying diagram. Both feed and coolant flows were maintained at a steady rate of 600 ± 5 mL/min using LOIP LS-301 peristaltic pumps (LOIP Ltd., Saint Petersburg, Russia), with flow rates assessed using rotameters. A detailed schematic of the entire system for AGMD distillation and assessment of the results is presented in Figure 1.

The droplets of permeate formed upon condensation on the coolant plate are directed by gravity into a measuring cylinder placed on the bottom of the membrane distillation module, allowing volumetric assessment of the collected permeate. The efficiency of the membrane distillation process was quantified using the salt rejection factor (R), calculated according to the appropriate formula for performance evaluation.

The salt rejection degree (R) was assessed by measuring the electrical conductivity on the permeate side during 6 h of MD operation using the following equation:

(2) $$R=\frac{Co-Cx}{Co}\times 100\%$$

where Co represents the concentration of salt in the feed solution (g/L) and Cx—concentration of salt in condensate (g/L). The salt concentration in the feed solution and condensate was determined conductometrically using the Starter 3100C device (OHAUS Co., Shanghai, China) [56].

## 3. Results and Discussion

### 3.1. Wettability Properties of the As-Obtained TFC Membranes (Water Contact Angle)

**Figure 2:**
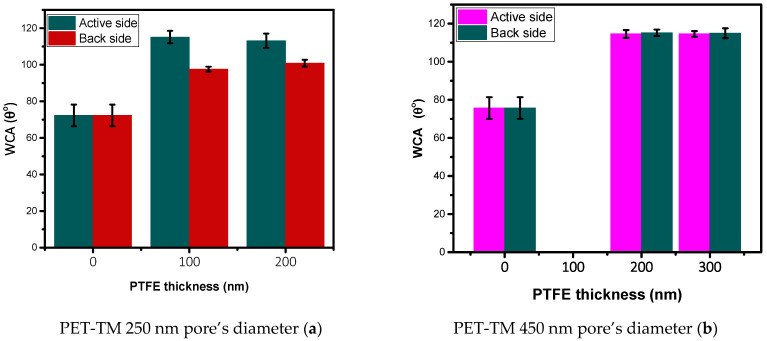
The dependence of the water contact angle on *active and back sides* of PET-TM/PTFE TFC membranes with initial effective pores diameters of 250 nm (**a**) and 450 nm (**b**).

The hydrophobic properties of the fabricated PET-TM/PTFE-like thin-film composite (TFC) membranes were initially evaluated, as this property is critical for effective operation in the Air Gap Membrane Distillation (AGMD) process. Wettability assessments of the initial PET-TM membranes and of the *active and back sides* of the PET-TM/PTFE-like thin film composite membranes are depicted in Figure 2.

The data illustrate the variation in water contact angles for both the initial PET-TM membranes and their composite counterparts as a function of PTFE-like coating thickness, highlighting the development of hydrophobic properties in these materials. Illustrative images of the water droplets on the PET-TM initial surface and PET-TM (250 nm)/PTFE-like (100 nm) on the *active side* and *back side* are presented in Figure S1 of the Supplementary Material. Notably, the *active side* of the tested TFC membranes exhibits a significant increase in water contact angle with values above 112°, demonstrating an enhanced hydrophobicity compared to the unmodified PET-TM membranes regardless of the initial pore diameters. This enhancement is consistent across all assessed PTFE-like thin film thicknesses. Contact angle measurements provide further validation of the hydrophobization of the PET-TM membranes on the *back side* of the PTFE-like coated TFC membranes. Although slightly lower than the WCA measured on the top surface, the *back side* also displays strong hydrophobic characteristics, confirming the effectiveness of the PTFE deposition process in modifying both membrane surfaces, most probably due to the energetic species resulting upon magnetron sputtering of the PTFE target. Effective hydrophobization was achieved on both sides of the composite membranes fabricated from the PET-TM substrate with a 450 nm pore diameter. While the *active side* of the 250 nm pore diameter membrane exhibits a water contact angle (WCA) comparable to that of the 450 nm membrane, the *back side* shows limited hydrophobicity relative to its *active side*, while the values are still consistent above 90°. The difference arises from the more limited diffusion of the fluorine-containing deposition species through the pore channels of lower diameter, accompanied by faster occlusion of membranes with smaller pore diameters during the PTFE-like deposition process. The dual-side enhancement of hydrophobicity achieved through PTFE-like coatings underscores the membranes’ suitability for air gap membrane distillation (AGMD) applications, where hydrophobicity is essential for maintaining efficiency. At the same time, it highlights the robustness of the PTFE-like coatings present on the *active side* together and suggests the formation of a fluorinated surface on the membrane’s *back side*, positioning these membranes as highly effective for extended use in distillation processes.

While the water contact angle at a surface is the key parameter that indicates which membrane is more hydrophobic, when using membranes in the processes of membrane distillation for water desalination, another important parameter is LEP_W_, which should be as high as possible in order to avoid the wetting of the pores. According to Equation (1), which is used to calculate LEP_W_, this value depends on the maximum pore size and the hydrophobicity of a membrane. Hence, to reach high LEP_W_ values, the membranes should be obtained from highly hydrophobic materials with low energy and should have a small pore size [57]. However, one should note that the choice of membranes with small pore sizes may cause a decrease in the productivity of membrane distillation because of low membrane permeability. In other words, to achieve high productivity, the membranes used must have high permeabilities and high LEP_W_ values. The LEP_W_ values for composite membranes obtained by depositing PTFE-like coatings with different thicknesses onto the surface of a PET-TM are listed in Table 1 (the pore radii are the maximum pore radii on the surface of composite membranes).

The presented data show that the membrane PET-TM 250 nm/PTFE 200 nm has the best salinity characteristics, and the pore diameter on the surface of this TFC is equal to 140 nm. This membrane requires more pressure to moisten its powers, although it might be inferior in performance to other composite membranes.

### 3.2. Morphological Properties of the As-Obtained TFC Membranes (Scanning Electron Microscopy)

**Figure 3:**
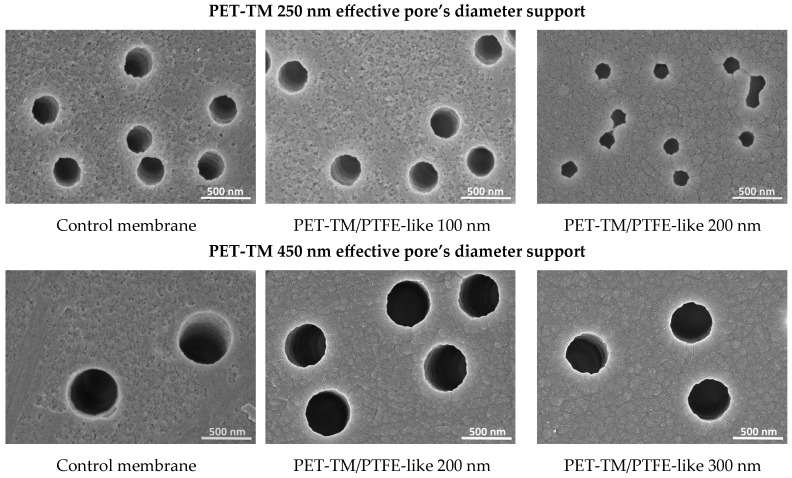
Top-view SEM images of the *active side* surface for both control PET-TM membranes with 250 nm and 450 nm initial effective pores diameter and the PET-TM/PTFE-like thin-film composite (TFC) membranes with various thicknesses of PTFE-like layers.

The presence of PTFE-like coatings on PET-TM membrane surface was confirmed through Scanning Electron Microscopy (SEM) analysis. Figure 3 presents SEM top-view images of the active surface for both control PET-TM membranes (250 nm and 400 nm pores diameter) and the PET-TM/PTFE thin-film composite (TFC) membranes. The SEM top view micrographs of the active surface of the as-investigated membranes illustrate a progressive occlusion of the pore structures correlating with the thickness of the PTFE layer. In contrast, this phenomenon is less pronounced on the *back side* (not presented here), where the pore diameter retains a size similar to that of the initial control membranes [52]. The morphology of the pores on the *active side* remains predominantly circular for both membrane types, with minor irregularities along the perimeter in the case of PET-TM 250 nm coated with a thicker PTFE layer, attributed to the characteristics of the deposited film. Only a marginal reduction in diameter is observed for the *back side* pores, which were not directly subjected to the magnetron sputtering deposition [52]. This indicates that during film deposition, a portion of the sputtered material infiltrates the pore openings and deposits on the internal walls, with prolonged deposition times potentially influencing the size of the *back side* pores as well.

### 3.3. Compositional Properties of the As-Obtained TFC Membranes (Fourier Infrared Spectroscopy and X-Ray Photoelectron Spectroscopy)

X-ray Photoelectron Spectroscopy (XPS) analysis provided detailed insights into the chemical mechanisms underlying the hydrophobization of PET-TM/PTFE-like Thin Film Composite (TFC) membranes via the surface chemistry modifications induced by the PTFE-like layer deposited through magnetron sputtering. These insights are essential for advancing the design and optimization of hydrophobic membranes tailored for Air Gap Membrane Distillation (AGMD) applications. Table 2 presents the atomic concentrations determined from the XPS survey spectra and the fluorine-to-carbon (F/C) ratios for all investigated PET-TM/PTFE-like TFC membranes. Figure S2 of the Supplementary Files provides the XPS survey spectra for the initial PET-TM membrane with 250 nm initial pores diameter and those of PET-TM/PTFE-like TFC membranes obtained from upon 100 nm, respectively 200 nm PTFE-like deposition.

**Figure 4:**
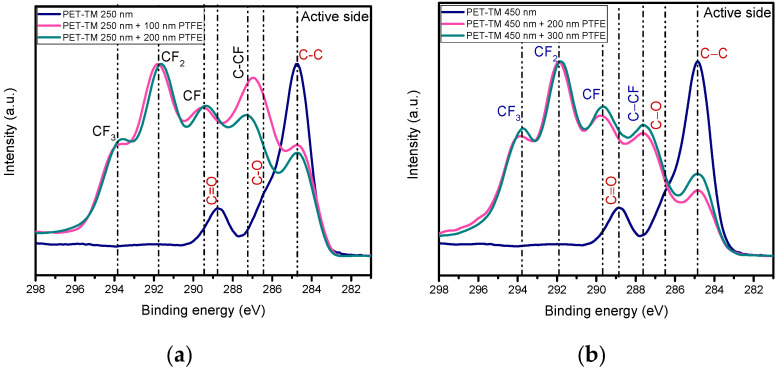
Comparative XPS high-resolution spectra of the C1s region for the *active side* of the initial PET-TM of (**a**) 250 nm and (**b**) 450 nm pores diameter and of PET-TM/PTFE-like TFC membranes with various thicknesses of the PTFE-like layer deposited on the *active side*.

The F/C ratio presented in Table 2 for the active surface of the PET-TM/PTFE TFC membranes ranges from approximately 2.5 to 3.6, depending on the thickness of the PTFE layer. Notably, the F/C ratios for all examined TFC membranes exceed the ratio reported for conventional PTFE (2.16), underscoring the effective retention of fluorine-based species released from the PTFE target during magnetron sputtering deposition. This observation also suggests minimal contamination from carbon in the natural environment and the presence of fluorine-based molecules trapped within the pores. In contrast, an analysis of the F/C ratio on the backside surface reveals a gradual increase, progressing from 0.07 for the PET-TM with a 250 nm initial pore size diameter coated with 100 nm of PTFE-like film to 0.38 for the membrane with a 200 nm PTFE-like coating. This trend indicates a more rapid occlusion of pore channels in membranes with a 250 nm pore diameter, reflecting a smooth transition from a carbon–oxygen-dominated surface associated with the PET-TM substrate to a fluorine-dominated surface. Additionally, the F/C value experiences a significant increase—by an order of magnitude—for membranes coated with a thicker PTFE layer. For the backside of the PET-TM membranes with a pore diameter of 450 nm, the F/C ratios are 0.47 for a 200 nm PTFE-like coating and increases up to 1.70 for a 300 nm PTFE-like coating, approaching the values found in conventional PTFE although no direct exposure of the membrane to the magnetron sputtering of PTFE was conducted. The photoelectron spectral analysis indicates that the control PET-TM membrane is predominantly composed of carbon and oxygen, whereas the as-synthesized TFC membranes show a significant presence of fluorine and carbon, with residual oxygen observed at the surface. The presence of oxygen is likely attributed to surface contamination during plasma deposition or X-ray-induced photoionization of electrons from the walls of the partially PTFE-coated membrane pores. As a result, the surface chemistry shifts towards hydrophobicity, evidenced by an increased contact angle, which is conducive to the membranes’ application in the Air Gap Membrane Distillation (AGMD) process. Figure 4 displays the overlay of high-resolution spectra for the C1s regions across the active surfaces of the membranes, with pore diameters of 250 nm and 450 nm. Comparative graphs of the C1s region of the *active side* of the as-synthesized TFC membranes, presented in Figure 4, reveal a progressive shift in bonding states. This transition, characterized by a decrease in C–O-based surface bonds and an increase in C–F interactions, becomes more pronounced as the thickness of the PTFE-like layer increases. This trend is consistently observed for PET-TM membranes with 250 nm and 450 nm pore sizes. A comprehensive analysis of the C1s region for the as-synthesized TFC membranes reveals three distinct peaks for the PET-TM porous support: C–C at 284.8 eV, C–O bond at 286.3 eV, and C=O bonds at 289 eV. In contrast, the TFC membranes display five distinct subpeaks, identified as follows: C–C at 284.8 eV, C–CF at 287.7 eV, C–F at 288.7 eV, and CF_2_ and CF_3_ bonds at 292 eV and 294 eV, respectively [52,53,58].

**Figure 5:**
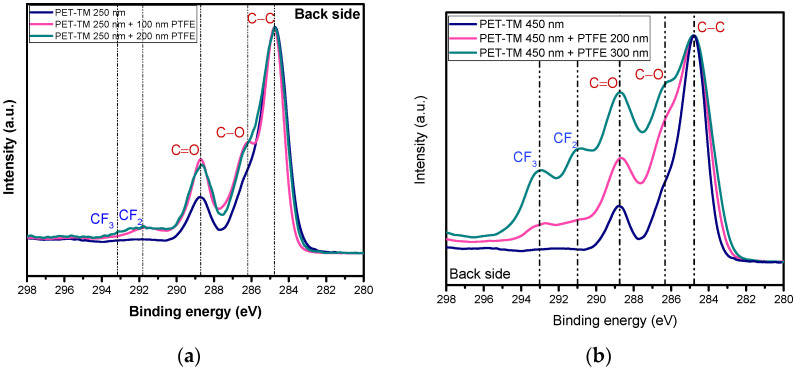
Comparative XPS high-resolution spectra of C1s region for the *back side* of the initial PET-TM with pores diameter of (**a**) 250 nm and (**b**) 450 nm and those resulting upon deposition on their *active side* of PTFE-like layers with various thicknesses.

Figure 5 displays the overlay of high-resolution spectra for the C1s regions across the *back side* of the TFC membranes, with pore diameters of 250 nm and 450 nm. Comparative graphs of the C1s region for the as-synthesized TFC membranes, presented in Figure 5, reveal a tendency of shifting of bonding states in the case of PET-TM membranes with pores diameter of 450 nm towards the PTFE-like chemical structure, especially for the 300 nm PTFE layer thickness. This transition, characterized by a decrease in the percentage of C–O-based surface bonds and an increase in those associated with C–F interactions, becomes more pronounced as the thickness of the PTFE layer increases. On the other hand, the TFC membranes synthesized using PET-TM with a pore diameter of 250 nm exhibit a much lighter transition toward C–F surface bonds, evidenced by the appearance of only minor contributions from CF_2_ and CF_3_ bonds in the overall C1s high-resolution spectra. Despite this limited presence of C–F bonds, the fluorine presence is evident, and the backside hydrophobization is observed, indicating the effectiveness of these contributions in altering surface properties towards a fluorinated surface.

**Figure 6:**
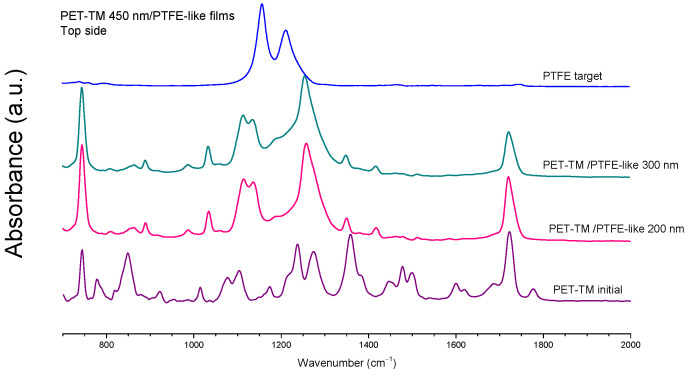
ATR-FTIR spectra of the *active side* for PET-TM 450 nm coated with 200 nm and 300 nm PTFE-like layers.

XPS measurements were corroborated with ATR-FTIR analyses performed on both the PET-TM membranes and PTFE-like coated PET-TM membranes with a pore size of 450 nm. Figure 6 presents the ATR-FTIR spectra of the PET-TM/PTFE-like thin-film composite membranes with coatings of 200 nm and 300 nm, compared to the control PET-TM sample (450 nm pore size). It should be highlighted from the beginning that the penetration depth of the IR radiation in the ATR-FTIR with germanium crystal is around 700 nm; therefore, the signal is a convolution of the bands originating from the original PET-TM membrane with those associated with PTFE-like coating. From the data provided, it follows that the deposition on the track-etched membrane surface leads to a change in the chemical composition of its surface layer.

The spectrum of the control membrane in the 2000–600 cm^−1^ wavenumber range is characterized by strong absorption bands associated with terephthalate groups (OCC_6_H_4_COO), which are prominently observed at 1120 cm^−1^ and 1244 cm^−1^. Intense bands at 1712 cm^−1^ and 1096 cm^−1^ correspond to the stretching vibrations of ester carbonyl groups and ester C–O groups, respectively. Additionally, the prominent band at 725 cm^−1^ is attributed to interactions between the polar ester group and the benzene ring. Weaker absorption bands are also present: the bands at 1585 and 1505 cm^−1^ are linked to aromatic skeleton vibrations involving C=C bond stretching. The bands at 1470, 1409, and 1340 cm^−1^ correspond to bending and wagging vibrations of the ethylene glycol segments, while those at 1017 and 871 cm^−1^ are associated with bending vibrations of aromatic rings. Moreover, the initial PET track-etched membrane exhibits weak bands at 971 and 847 cm^−1^, corresponding to stretching vibrations of C–O bonds and rocking vibrations of methylene groups. In the TFC membranes, key absorption bands typical of magnetron-sputtered PTFE-like films were identified: CF=O vibrations at 725 cm^−1^, imposed to the original band of PET-TM membrane but wider and with higher intensity, CF_3_ stretching at 978 cm^−1^, CF_2_ stretching at 1182 cm^−1^ and 1227 cm^−1^, along with combined CF_2_ asymmetric stretching and rocking deformations at 1407 cm^−1^. Additionally, C–O group bands from the PET-TM structure create overlap at 1715 cm^−1^, attributed to C–CF_2_ or CF=CF_2_ vibrations, underscoring the cross-linking characteristic of the deposited films [50,59]. The spectra also indicate alterations in surface chemistry on both membrane sides in correlation with increased PTFE deposition time. Notably, the CF_2_ peak demonstrates a significant retention of PTFE-like bonds within the deposited film, with a more pronounced increase in the CF_2_ contribution on the *active side* of the composite membrane. However, original PTFE chemical bonds undergo degradation during the magnetron sputtering process, as evidenced by the CF_3_ peak, reflecting significant carbon bond termination in the resulting polymer fragments. In other words, the structure and properties of the polymer coatings deposited by magnetron sputtering of PTFE differ significantly from the structure and properties of the polymer obtained using conventional chemical polymerization processes, justifying the use of the term “PTFE-like” to refer to the deposited polymer in order to emphasize these differences with respect to the well known PTFE material. In conclusion, the spectra reveal additional IR peaks corresponding to fluorine functional groups compared to the PET-TM control, confirming the formation of PTFE-like layers on the membrane’s top surface. Moreover, chemical modifications on the non-exposed side (*back side*) suggest significant penetration of fluorine plasma species into the PET-TM support material, altering the inner surfaces down to the substrate holder level.

### 3.4. Water Transport in the Membrane Distillation Process

Table 3 presents a comprehensive analysis comparing the properties and performance of initial PET-TM membranes with pore sizes diameter of 250 nm and 450 nm, alongside their composite variants enhanced by PTFE-like coatings applied via magnetron sputtering. The findings indicate noteworthy trends in pore architecture, porosity, flux, and separation efficiency, highlighting the inherent trade-offs between flux and selectivity in membrane distillation applications. Deposition of PTFE-like coatings leads to a progressive reduction in both effective and surface pore diameters. For membranes with a 250 nm pore size, the effective pore diameter diminishes to 215 nm upon the deposition of the thickest PTFE-like coating. Conversely, 450 nm membranes exhibit a reduction in effective pore diameter to 375 nm. Additionally, increasing the PTFE-like layer thickness correlates with a decrease in porosity, which declines from 9.8% to 7.3% for 250 nm membranes and from 9.5% to 6.6% for the 450 nm counterparts.

In terms of performance, membranes with larger pore diameters demonstrate higher maximum condensate flow rates. The uncoated 450 nm membranes achieve flow rates of 4950 g/m^2^·h, while the uncoated 250 nm membranes reach 1815 g/m^2^·h without displaying relevant salt rejection properties. However, the introduction of PTFE-like coatings resulted in a marked reduction in flux for both types of TFC membranes, yielding 1175 g/m^2^·h for the 450 nm membranes and 970 g/m^2^·h for the 250 nm versions at maximum coating thickness. Regarding salt rejection, uncoated 250 nm membranes show a rate of 53.45%, whereas 450 nm membranes achieve only 31.65%. After applying PTFE-like coatings, both membrane variants exhibit rejection rates surpassing 99%, with the smaller pore membranes displaying marginally better performances. Specifically, the PET-TM250 nm/PTFE-like coating of 200 nm membranes, with a 215 nm effective pore diameter, yields a salt concentration of 55.1 mg/L, corresponding to a salt rejection rate of 99.63% and conductivity of 116.9 µS/cm. Instead, the 450 nm membranes, with a 375 nm effective pore diameter corresponding to 300 nm PTFE-like coating, show higher salt concentrations (78.5 mg/L) corresponding to a salt rejection of 99.47% and conductivity of 165.7 µS/cm. Furthermore, the gas permeability reduction induced by the PTFE-like plasma coating enhances the separation efficiency of these membranes. Composite membranes consistently achieve salt rejection rates exceeding 99%, with retention coefficients (R) ranging from 99.06% to 99.63%. In stark contrast, uncoated PET-TM membranes demonstrate suboptimal desalination performance, with retention rates of only 53.45% for the 250 nm pores membranes and even lower, down to 31.45% for the 450 nm variant. Remarkably, the PET-TM membrane of 450 nm pore diameter with a 300 nm PTFE-like coating achieves an average salt concentration reduction of more than 190 times when treating a 15 g/L sodium chloride solution. The analysis underscores the importance of balancing pore size, porosity, and PTFE-like coating thickness to optimize flux and selectivity in membrane distillation. While larger pore sizes provide higher flux, they compromise the salt rejection. Conversely, smaller pore sizes, especially those below 240 nm, ensure superior desalination performance with lower conductivity and salt concentrations in the condensate. These findings demonstrate the critical role of membrane design in achieving high efficiency in desalination applications.

### 3.5. Temporal Performance of PET-TM/PTFE TFC Membranes in the AGMD Process

**Figure 7:**
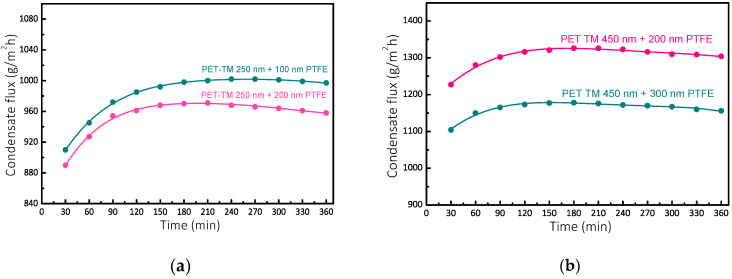
Dependence of condensate flux in time for PET track-etched membrane with a pore diameter of 250 nm (**a**) and 450 nm (**b**) after deposition of PTFE-like layers with various thicknesses.

The performance of PET-TM/PTFE thin-film composite (TFC) membranes in salt separation via air gap membrane distillation (AGMD) is summarized in Figure 7, which illustrates the temporal productivity trends.

A significant initial increase in productivity was observed across all membrane configurations, slightly peaking before gradually declining but still remaining at high values. Among the evaluated TFC membranes, PET-TM membranes with a 450 nm pore diameter and PTFE-like coatings of 200 nm and 300 nm thickness demonstrated the highest condensate flow rates, reaching 1325 ± 5 g/m^2^·h and 1175 ± 5 g/m^2^·h, respectively. In contrast, PET-TM membranes with a 250 nm pore size and PTFE-like coatings of 100 nm and 200 nm thickness exhibited lower peak flow rates of 1005 ± 5 g/m^2^·h and 970 ± 5 g/m^2^·h, respectively. In a comparative analysis, a commercial polyvinylidene fluoride (PVDF) membrane (Millipore, Taufkirchen, Germany) exhibiting a pore size of 450 nm demonstrated a flux rate of 1200 g/m^2^·h during the desalination process of a 15 g/L sodium chloride solution at a temperature of 50 °C, while the coolant temperature was consistently maintained at 10 °C [60]. The results highlight the enhanced performance of the PET-TM 450 nm/PTFE 200 nm thin-film composite (TFC) membranes, which demonstrated significantly higher flux rates than the investigated commercial PVDF membrane [60]. Key operational parameters, including the temperatures of the heating and cooling fluids, the concentration of feed salt, the width of the air gap, and the flow velocities, substantially affect the condensate flux [61]. Moreover, the characteristics of the membrane, particularly pore size and the configuration of the distillation module, played critical roles in the overall performance of the TFC systems [62]. Implementing reduced pore diameters in TFC membranes resulted in improved separation efficiency, effectively offsetting the decline in flux observed. Operational stability of our membranes showed that condensate flow rates stabilized after 90–120 min runtime, maintaining a steady state distillation process for an additional four hours with only a negligible reduction of 0.02% from the peak flow rate recorded. Regarding durability, the prolonged operation was linked to a slight decrease in productivity attributed to partial degradation of the PTFE-like layer, which potentially allowed for the permeation of byproducts into the membrane pores. The reduction in the condensate flow with time could also be caused by salt deposition onto the working surface of a membrane, i.e., its fouling. The study of the wetting of the TFC membranes after the end of the MD process has shown that the water contact angle at membrane surfaces insignificantly decreases by only 4−6° depending on the coating thickness. This may indicate the partial degradation of the polymer layer. However, analogous variations in the condensate flow with time are also observed for the initial PET and PP track-etched membranes. As we have shown in [56], these membranes also exhibit an increase in the condensate flow to a certain maximum value at the initial stage, followed by a gradual decrease in the flow value. Therewith, the initial PET-TM has exhibited the maximum value of the condensate flow equal to 1815 ± 10 g/m^2^·h, attributed to the hydrophilic properties of the membrane matrix. Instead, for the PP membrane that exhibits hydrophobic properties, the maximum flow through it was as low as 630 ± 4 g/m^2^·h. The considerably lower value was attributed to its larger thickness compared to that of the hydrophobic PTFE-like coating of the composite membranes described in this work. The decrease in the condensate flow with time for the initial TMs leads us to suppose that the main reason for the reduction in the separation productivity of the composite membranes is, most probably, the design of the membrane separating module rather than the degradation of the PTFE-like coating. The membranes exhibited significant hydrophobicity, highlighting their potential for long-term use in desalination applications. This follows the latest developments in the field of fluoropolymer-based membranes [63], which evidenced that the available membranes still need improvements with respect to their hydrophobic character and the diminution of traditional chemical solvents used. The proposed method of using plasma sputtering of PTFE avoids any solvent while inducing significant hydrophobicity to the conventional and much cheaper PET material. The advantages of a composite architecture were evident, with dual-layer composite membranes showing improved performance metrics.

The hydrophobic PTFE-like layer greatly enhanced vapor transport efficiency, while the hydrophilic substrate effectively minimized hydraulic resistance, optimizing desalination’s overall efficacy. These findings highlight the importance of balancing pore size, PTFE-like layer thickness, and structural design to achieve optimal efficacy in membrane distillation processes.

## 4. Conclusions

RF magnetron sputtering has proven to be an effective plasma-based technique for creating hydrophobic thin film composite PET-TM/PTFE-like membranes suitable for Air Gap Membrane Distillation (AGMD). This deposition method allows for precise control over TFC membrane properties, such as pore size, surface hydrophobicity, and structural integrity, which leads to improved desalination performance. The PTFE-like coated PET-TM membranes exhibited enhanced hydrophobicity on both sides, making them ideal for AGMD, where hydrophobicity is crucial for efficient operation. Scanning Electron Microscopy (SEM) analysis confirmed the successful application of PTFE-like coatings on the PET-TM membranes, revealing changes in pore morphology that correlated with the thickness of the PTFE-like layer. The active surface displayed progressive pore occlusion as the PTFE-like layer thickened, while on the *back side*, the modification of the pore diameters was less intense, especially in the case of PET-TM membranes with initial effective pores diameter of 250 nm. X-ray Photoelectron Spectroscopy (XPS) analysis further validated the hydrophobization process, showing the significant fluorine content on both sides of membranes’ surfaces compared to the original PET-TM composition, including only carbon and oxygen. This shift in surface chemistry, characterized by increased C–F related bonds and reduced C–O bonds, was linked to enhanced hydrophobicity, as indicated by increased contact angles. Attenuated Total Reflectance-Fourier Transform Infrared Spectroscopy (ATR-FTIR) analysis also confirmed the chemical modifications induced by the PTFE-like coating on the *active side*, identifying the apparition of specific PTFE-related absorption bands that coexist with those of the initial PET-TM membrane due to the penetration depth of ATR-FTIR technique. In terms of performance, the PTFE-like coated PET-TM membranes demonstrated improved salt rejection but a decrease in flux. Applying a PTFE-like layer reduced pore diameter and membrane porosity, TFC membranes based on PET-TM with smaller pore membranes (250 nm) exhibited better desalination efficiency and lower salt concentrations in the condensate. These results highlight the trade-off between flux and selectivity, underscoring the importance of optimizing membrane design-balancing pore size, porosity, and PTFE-like coating thickness to maximize efficiency in desalination applications. Overall, PET-TM membranes with PTFE-like coatings displayed superior performance, particularly the 450 nm pore size with a 200 nm PTFE coating, which achieved higher flux rates than commercial PVDF membranes. Although flux slightly decreased over time due to partial PTFE degradation, the membranes maintained high hydrophobicity and stability, making them suitable for long-term use. The durability of the hydrophobic properties further confirms these membranes’ potential for long-term applications in desalination and wastewater treatment. By balancing flux and salt rejection through optimal membrane design, we have shown that it is possible to develop high-performance membranes that address challenges in sustainable water management. Further experiments will be performed in order to make a complete assessment of the membranes’ applicative potential under various operating temperatures and pHs, as well as their chemical stability and surface fouling under long-term operation. Such an approach will allow a better understanding of the mass transfer mechanisms during the AGMD process.

## Figures and Tables

**Table 1:** Liquid entry pressures of the TFC membranes with respect to initial pore diameter and PTFE thickness.

PET-TM/PTFE-like TFC Membranes	LEP_W_ (kPa)
PET-TM 250 nm/PTFE 100 nm	282
PET-TM 250 nm/PTFE 200 nm	432
PET-TM 450 nm/PTFE 200 nm	217
PET-TM 450 nm/PTFE 300 nm	255

**Table 2:** Elemental concentration at the surface of PET-TM/PTFE, such as TFC membranes and F/C ratios, is used for various combinations of membranes’ pores and thicknesses of PTFE-like thin films.

PET-TM/PTFE-like TFC Membranes *active side*				
TFC membranes/Element	C1s	O1s	F1s	F/C Ratio
PET-TM 250 nm/PTFE 100 nm	24.03	3.07	72.89	3.03
PET-TM 250 nm/PTFE 200 nm	20.59	3.71	75.71	3.68
PET-TM 450 nm/PTFE 200 nm	28.02	2.65	69.33	2.47
PET-TM 450 nm/PTFE 300 nm	27.21	2.69	70.10	2.58
**PET-TM/PTFE-like TFC Membranes *back side***				
**TFC membranes/Element**	**C1s**	**O1s**	**F1s**	**F/C Ratio**
PET-TM 250 nm/PTFE 100 nm	68.84	26.33	4.83	0.07
PET-TM 250 nm/PTFE 200 nm	60.80	28.36	10.84	0.38
PET-TM 450/PTFE 200 nm	53.27	21.58	25.16	0.47
PET-TM 450 nm/PTFE 300 nm	32.30	12.70	54.99	1.70

**Table 3:** Comparison of PET-TM 250 nm and 450 nm and composite membranes performance and selectivity of separation in the process of membrane distillation.

PET-TM/PTFE Composite Membranes	PET-TM 250			PET-TM 450		
	Thickness of the PTFE Layer, nm					
	Control	100	200	Control	200	300
Effective pore diameter, nm	250	240	215	450	400	375
Surface pore diameter, nm	295	250	140	500	415	390
Porosity, %	9.8	9.0	7.3	9.5	7.5	6.6
Maximum condensate flow, g/m^2^·h	1815	1005	970	4950	1325	1175
Condensate electrical conductivity, μCm/cm	12,800	293.8	116.9	18,500	196.2	165.7
Concentration of salt in the condensate, mg/L	6980	140.5	55.1	10,250	92.1	78.5
Salt rejection factor (R), %	53.45	99.06	99.63	31.65	99.35	99.47

## Data Availability

The original contributions presented in this study are included in this article; further inquiries can be directed to the corresponding authors.

## Supplementary Materials

The following supporting information can be downloaded at https://www.mdpi.com/article/10.3390/polym17030290/s1, Figure S1. Examples of water contact angle images of PET-TM (250 nm) membranes (a) initial; (b) *active side* and (c) *back side* of PET-TM (250 nm)/PTFE-like coated with 100 nm layer; Figure S2. XPS survey spectra on initial PET TM membrane with 250 nm effective pores diameter and those of *active side* of PET-TM250/PTFE-like TFC membranes with 100 nm and 200 nm thickness.

## References

[1] United Nations Environment Programme (UNEP) (2006). Challenges to International Waters—Regional Assessments in a Global Perspective.

[2] World Water Assessment Programme (WWAP) (2012). The United Nations World Water Development Report 4: Managing Water under Uncertainty and Risk.

[3] World Water Assessment Programme (WWAP) (2015). The United Nations World Water Development Report 2015: Water for a Sustainable World.

[4] UNESCO (2021). Valuing Water, The United Nations World Water Development Report 2021.

[5] UN-Water (2023). Summary of Proceedings—UN 2023 Water Conference. In Proceedings of the 2023 UN Water Conference, New York, NY, USA, 22–24 March 2023.

[6] Elimelech M., Phillip W.A. (2011). The Future of Seawater Desalination: Energy, Technology, and the Environment. Science.

[7] Al-Othman A., Darwish N.N., Qasim M., Tawalbeh M., Darwish N.A., Hilal N. (2019). Nuclear Desalination: A State-of-the-Art Review. Desalination.

[8] Bonyadi S., Chung T.S. (2007). Flux Enhancement in Membrane Distillation by Fabrication of Dual Layer Hydrophilic–Hydrophobic Hollow Fiber Membranes. J. Membr. Sci..

[9] Gryta M. (2007). Influence of Polypropylene Membrane Surface Porosity on the Performance of Membrane Distillation Process. J. Membr. Sci..

[10] Wei X., Zhao B., Li X.-M., Wang Z., He B.-Q., He T., Jiang B. (2012). CF4 Plasma Surface Modification of Asymmetric Hydrophilic Polyethersulfone Membranes for Direct Contact Membrane Distillation. J. Membr. Sci..

[11] Rastegarpanah A., Mortaheb H.R. (2016). Surface Treatment of Polyethersulfone Membranes for Applying in Desalination by Direct Contact Membrane Distillation. Desalination.

[12] Okiel K., El-Aassar A.H.M., Temraz T., El-Etriby S., Shawky H.A. (2016). Performance Assessment of Synthesized CNT/Polypropylene Composite Membrane Distillation for Oil Field Produced Water Desalination. Desal. Water Treat..

[13] Leaper S., Abdel-Karim A., Gad-Allah T.A., Gorgojo P. (2019). Air-Gap Membrane Distillation as a One-Step Process for Textile Wastewater Treatment. Chem. Eng. J..

[14] Eykens L., Hitsov I., De Sitter K., Dotremont C., Pinoy L., Van der Bruggen B. (2017). Direct Contact and Air Gap Membrane Distillation: Differences and Similarities Between Lab and Pilot Scale. Desalination.

[15] Alkhudhiri A., Darwish N., Hilal N. (2012). Membrane Distillation: A Comprehensive Review. Desalination.

[16] Misdan N., Ismail A.F., Hilal N. (2016). Recent Advances in the Development of (Bio)fouling Resistant Thin Film Composite Membranes for Desalination. Desalination.

[17] Bhadra M., Roy S., Mitra S. (2016). Flux Enhancement in Direct Contact Membrane Distillation by Implementing Carbon Nanotube Immobilized PTFE Membrane. Sep. Purif. Technol..

[18] Eykens L., Hitsov I., De Sitter K., Dotremont C., Pinoy L., Nopens I., Vander Bruggen B. (2016). Influence of Membrane Thickness and Process Conditions on Direct Contact Membrane Distillation at Different Salinities. J. Membr. Sci..

[19] Naidu G., Jeong S., Vigneswaran S., Jang E.-K., Choi Y.-J., Hwang T.-M. (2016). Fouling Study on Vacuum-Enhanced Direct Contact Membrane Distillation for Seawater Desalination. Desal. Water Treat..

[20] Luo M.-L., Zhao J.-Q., Tang W., Pu C.-S. (2005). Hydrophilic Modification of Poly(ether sulfone) Ultrafiltration Membrane Surface by Self-Assembly of TiO_2_ Nanoparticles. Appl. Surf. Sci..

[21] Pandele A.M., Comanici F.E., Carp C.A., Miculescu F., Voicu S.I., Thakur V.K., Serban B.C. (2017). Synthesis and Characterization of Cellulose Acetate-Hydroxyapatite Micro and Nano Composites Membranes for Water Purification and Biomedical Applications. Vacuum.

[22] Voicu S.I., Ninciuleanu C.M., Muhulet O., Miculescu M. (2014). Cellulose Acetate Membranes with Controlled Porosity and Their Use for the Separation of Amino Acids and Proteins. J. Optoelectron. Adv. Mater..

[23] Muscalu C., David R., Garea S.A., Nechifor A.C., Vaireanu D.I., Voicu S.I., Nechifor G. Polysulfone-Polypyrrole Ionic Conductive Composite Membranes Synthesized by Phase Inversion with Chemical Reaction. Proceedings of the 2009 International Semiconductor Conference.

[24] Shannon A.M., Bohn W.P., Elimelech M., Georgiadis J.G., Mariñas B.J., Mayes A.M. (2008). Science and technology for water purification in the coming decades. Nature.

[25] Makkonen-Craigi S., Yashina K., Paronen M. (2014). Track-Etched Ultrafiltration Polymer Membranes Produced by Light Ion Irradiation. Arcada Work. Pap..

[26] Makkonen-Craigi S., Paronenii M. (2014). Potential Large-Scale Applications of Track-Etched Ultrafiltration Polymer Membranes. Arcada Work. Pap..

[27] Tai S.L., Abidin M.N.Z., Ma’amor A., Hashim N.A., Hashim M.L.H. (2025). Polyethylene Terephthalate Membrane: A Review of Fabrication Techniques, Separation Processes, and Modifications. Sep. Purif. Technol..

[28] Yeszhanov A.B., Korolkov I.V., Güven O., Melnikova G.B., Dosmagambetov S.S., Borissenko A.N., Nurkassimov A.K., Kassymzhanov M.T., Zdorovets M.V. (2024). Effect of Hydrophobized PET TeMs Membrane Pore-Size on Saline Water Treatment by Direct Contact Membrane Distillation. RSC Adv..

[29] Kholodovych V., Welsh W.J., Mark J.E. (2007). Thermal-Oxidative Stability and Degradation of Polymers. Physical Properties of Polymers Handbook.

[30] Huang J., Martinez-Vega J., Malec D. Morphological Evolution of Polytetrafluoroethylene (PTFE) during Thermal-Oxidative Ageing above and below the Melting Temperature. Proceedings of the 2013 IEEE International Conference on Solid Dielectrics (ICSD).

[31] Shakayeva A.K., Yeszhanov A.B., Zhumazhanova A.T., Korolkov I.V., Zdorovets M.V. (2024). Fabrication of Hydrophobic PET Track-Etched Membranes Using 2,2,3,3,4,4,4-Heptafluorobutyl Methacrylate for Water Desalination by Membrane Distillation. Eurasian J. Chem..

[32] Kravets L.I., Yarmolenko M.A., Rogachev A.A., Gainutdinov R.V., Gilman A.B., Altynov V.A., Lizunov N.E. (2020). Formation of Superhydrophobic Coatings on the Track-Etched Membrane Surface by the Method of Electron-Beam Deposition of Polymers in Vacuum. Inorg. Mater. Appl. Res..

[33] Wilde W. (1974). Evaporation of Polytetrafluoroethylene by Electron Bombardment of the Bulk Material. Thin Solid Film..

[34] Rastogi A.C., Desu S.B. (2005). Thermal Chemical Vapor Deposition of Fluorocarbon Polymer Thin Films in a Hot Filament Reactor. Polymer.

[35] Limb S.J., Labelle C.B., Gleason K.K., Edell D.J., Gleason E.F. (1996). Growth of Fluorocarbon Polymer Thin Films with High CF_2_ Fractions and Low Dangling Bond Concentrations by Thermal Chemical Vapor Deposition. Appl. Phys. Lett..

[36] Ohnishi Y., Kita R., Tsuchiya K., Iwamori S. (2016). Optical Characteristics of Poly(Tetrafluoroethylene) Thin Film Prepared by a Vacuum Evaporation. Jpn. J. Appl. Phys..

[37] Yi N., Bao S., Zhou H., Wang Q., Yang J., Xie X. (2016). Preparation of Microstructure-Controllable Superhydrophobic Polytetrafluoroethylene Porous Thin Film by Vacuum Thermal-Evaporation. Front. Mater. Sci..

[38] Biederman H. (2000). Organic Films Prepared by Polymer Sputtering. J. Vac. Sci. Technol. A.

[39] Norton M.G., Jiang W., Dickinson J.T., Hipps K.W. (1996). Pulsed Laser Ablation and Deposition of Fluorocarbon Polymers. Appl. Surf. Sci..

[40] Blanchet G.B., Shah S.I. (1993). Deposition of Polytetrafluoroethylene Films by Laser Ablation. Appl. Phys. Lett..

[41] Ju Y., Ai L., Qi X., Song W., Ai L. (2023). Review on Hydrophobic Thin Films Prepared Using Magnetron Sputtering Deposition. Materials.

[42] Huang F., Wei Q., Liu Y., Zhang L., Wang Y. (2007). Surface Functionalization of Silk Fabric by PTFE Sputter Coating. J. Mater. Sci..

[43] Bodas D.S., Mandale A.B., Gangal S. (2005). Deposition of PTFE Thin Films by RF Plasma Sputtering on Silicon Substrates. Appl. Surf. Sci..

[44] Zhang Y., Yang G.H., Kang E.T., Neoh K.G., Huang W., Huan A.C.H., Wu S.Y. (2002). Deposition of Fluoropolymer Films on Si(100) Surfaces by Rf Magnetron Sputtering of Poly(tetrafluoroethylene). Langmuir.

[45] Iwamori S., Hasegawa N., Uemura A., Tanabe T., Nishiyama I. (2009). Friction and Adhesion Properties of Fluorocarbon Polymer Thin Films Prepared by Magnetron Sputtering. Vacuum.

[46] Yang G.H., Zhang Y., Kang E.T., Neoh K.G. (2003). Deposition of Ultrathin Fluoropolymer Films on Si(100) and GaAs(100) Surfaces by RF Magnetron Sputtering of Poly(tetrafluoroethylene-co-hexafluoropropylene). J. Phys. Chem. B.

[47] Biederman H., Zeuner M., Zalman J., Bílková P., Slavínská D., Stelmasuk V., Boldyreva A. (2001). Rf Magnetron Sputtering of Polytetrafluoroethylene under Various Conditions. Thin Solid Film..

[48] Kravets L., Dmitriev S., Lizunov N., Satulu V., Mitu B., Dinescu G. (2010). Properties of Poly(ethylene Terephthalate) Track Membranes with a Polymer Layer Obtained by Plasma Polymerization of Pyrrole Vapors. Nucl. Instrum. Methods Phys. Res. Sect. B.

[49] Elinson V.M., Shchur P.A., Deshevaya E.A., Kravets L.I. (2019). Antimicrobial Antiadhesive Properties of Nanostructured Fluorocarbon Films Obtained under Transient Conditions Using Two-Component Gas Mixtures. J. Phys. Conf. Ser..

[50] Satulu V., Mitu B., Altynov V.A., Lizunov N.E., Kravets L., Dinescu G. (2017). Synthesis and Characterization of Porous Composite Membranes with Hydrophilic/Hydrophobic Sides. Thin Solid Film..

[51] Apel P.Y., Dmitriev S.N. (2011). Micro- and Nanoporous Materials Produced Using Accelerated Heavy Ion Beams. Adv. Nat. Sci. Nanosci. Nanotechnol..

[52] Satulu V., Mitu B., Pandele A.M., Voicu S.I., Kravets L., Dinescu G. (2019). Composite Polyethylene Terephthalate Track Membranes with Thin Teflon-Like Layers: Preparation and Surface Properties. Appl. Surf. Sci..

[53] Satulu V., Pandele A.M., Ionica G.-I., Bobirică L., Bonciu A.F., Scarlatescu A., Bobirică C., Orbeci C., Voicu S.I., Mitu B. (2024). Robust CA-GO-TiO_2_/PTFE Photocatalytic Membranes for the Degradation of the Azithromycin Formulation from Wastewaters. Polymers.

[54] Mulder M. (1996). Basic Principles of Membrane Technology.

[55] Rezaei M., Warsinger D.M., Lienhard V.J.H., Duke M.C., Matsuura T., Samhaber W.M. (2018). Wetting Phenomena in Membrane Distillation: Mechanisms, Reversal, and Prevention. Water Res..

[56] Kravets L.I., Yarmolenko M.A., Rogachev A.V., Gainutdinov R.V., Altynov V.A., Lizunov N.E. (2022). Formation of Hydrophobic and Superhydrophobic Coatings on Track-Etched Membrane Surfaces to Create Composite Membranes for Water Desalination. Colloid J..

[57] Khayet M., Matsuura T. (2001). Preparation and Characterization of Polyvinylidene Fluoride Membranes for Membrane Distillation. Ind. Eng. Chem. Res..

[58] Hubert J., Mertens J., Dufour T., Vandencasteele N., Reniers F., Viville P., Lazzaroni R., Raes M., Terryn H. (2015). Synthesis and Texturization Processes of (Super)-Hydrophobic Fluorinated Surfaces by Atmospheric Plasma. J. Mater. Res..

[59] Satulu V., Ionita M.D., Vizireanu S., Mitu B., Dinescu G. (2016). Plasma Processing with Fluorine Chemistry for Modification of Surfaces Wettability. Molecules.

[60] Hawlader M., Bahar R., Ng K.C., Wei L.J.S. (2012). Transport Analysis of an Air Gap Membrane Distillation (AGMD) Process. Desalination Water Treat..

[61] Khayet M., Mengual J.I., Matsuura T. (2005). Porous Hydrophobic/Hydrophilic Composite Membranes: Application in Desalination Using Direct Contact Membrane Distillation. J. Membr. Sci..

[62] Khayet M., García Payo C. (2016). Progress on Membrane Distillation and Related Technologies. J. Membr. Sci. Res..

[63] Li X., Pan J., Macedonio F., Ursino C., Carraro M., Bonchio M., Drioli E., Figoli A., Wang Z., Cui Z. (2022). Fluoropolymer Membranes for Membrane Distillation and Membrane Crystallization. Polymers.

