# Supplementary material for: High-Performance PET-TM/PTFE-like Composite Membranes for Efficient Salt Rejection via Air Gap Membrane Distillation
Source: Polymers (Basel). 2025 Jan 23;17(3):290. doi: 10.3390/polym17030290 (PMC11820516; doi:10.3390/polym17030290)
Supplement: Supplementary file 1 [file polymers-17-00290-s001.zip › polymers-3419408-supplementary.pdf]

Supplementary file of manuscript polymers - 3419408

*Article*

# High-Performance PET-TM/PTFE-like Composite Membranes for Efficient Salt Rejection via Air Gap Membrane Distillation

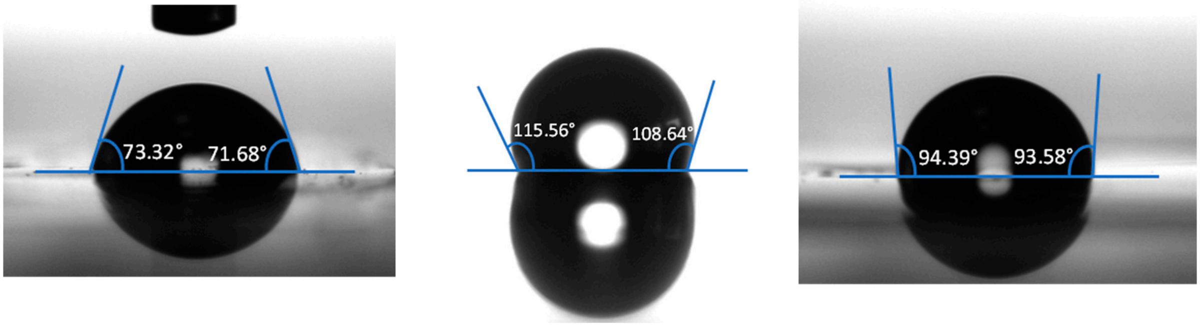


a) b) c)

**Figure S1.** Examples of water contact angle images of PET-TM (250 nm) membranes a) initial; b) *active side* and c) *back side* of PET-TM (250nm)/PTFE-like coated with 100 nm layer

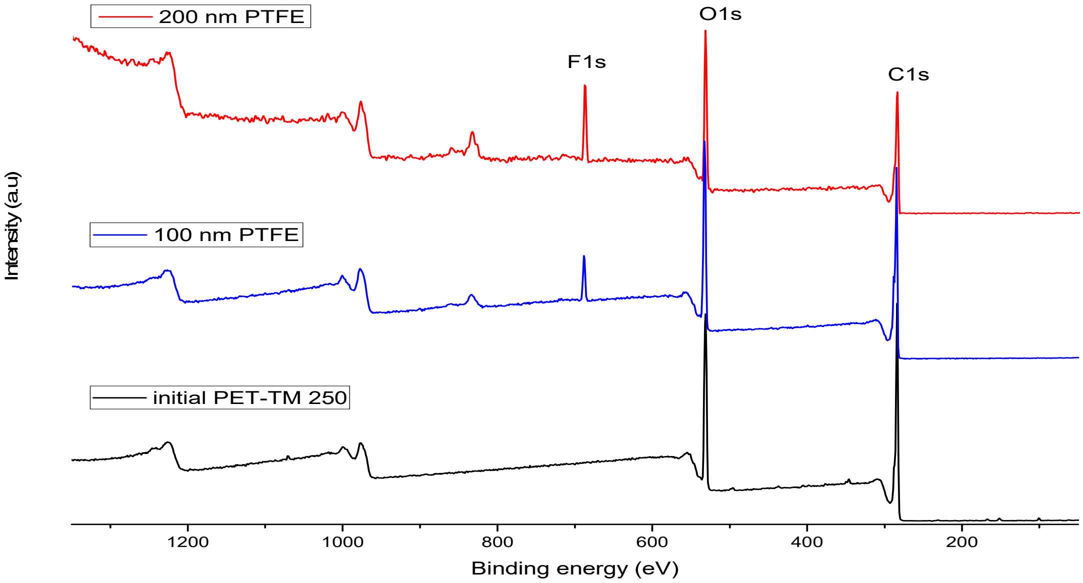


Figure S2: XPS survey spectra on initial PET TM membrane with 250 nm effective pores diameter and those of *active side* of PET-TM250/PTFE-like TFC membranes with 100 nm and 200 nm thickness.
